# High fidelity simulation of the endoscopic transsphenoidal approach: Validation of the UpSurgeOn TNS Box

**DOI:** 10.3389/fsurg.2022.1049685

**Published:** 2022-12-06

**Authors:** Nicola Newall, Danyal Z. Khan, John G. Hanrahan, James Booker, Anouk Borg, Joseph Davids, Federico Nicolosi, Siddharth Sinha, Neil Dorward, Hani J. Marcus

**Affiliations:** ^1^ Department of Neurosurgery, National Hospital for Neurology and Neurosurgery, London, United Kingdom; ^2^ Wellcome/EPSRC Centre for Interventional and Surgical Sciences (WEISS), London, United Kingdom; ^3^ Department of Surgery and Cancer, Imperial College London, London, United Kingdom; ^4^ School of Medicine and Surgery, University of Milano-Bicocca, Monza, Italy

**Keywords:** endoscopic, simulation, high fidelity, transsphenoidal approach, UpSurgeOn

## Abstract

**Objective:**

Endoscopic endonasal transsphenoidal surgery is an established technique for the resection of sellar and suprasellar lesions. The approach is technically challenging and has a steep learning curve. Simulation is a growing training tool, allowing the acquisition of technical skills pre-clinically and potentially resulting in a shorter clinical learning curve. We sought validation of the UpSurgeOn Transsphenoidal (TNS) Box for the endoscopic endonasal transsphenoidal approach to the pituitary fossa.

**Methods:**

Novice, intermediate and expert neurosurgeons were recruited from multiple centres. Participants were asked to perform a sphenoidotomy using the TNS model. Face and content validity were evaluated using a post-task questionnaire. Construct validity was assessed through post-hoc blinded scoring of operative videos using a Modified Objective Structured Assessment of Technical Skills (mOSAT) and a Task-Specific Technical Skill scoring system.

**Results:**

Fifteen participants were recruited of which *n* = 10 (66.6%) were novices and *n* = 5 (33.3%) were intermediate and expert neurosurgeons. Three intermediate and experts (60%) agreed that the model was realistic. All intermediate and experts (*n* = 5) strongly agreed or agreed that the TNS model was useful for teaching the endonasal transsphenoidal approach to the pituitary fossa. The consensus-derived mOSAT score was 16/30 (IQR 14–16.75) for novices and 29/30 (IQR 27–29) for intermediate and experts (*p* < 0.001, Mann–Whitney *U*). The median Task-Specific Technical Skill score was 10/20 (IQR 8.25–13) for novices and 18/20 (IQR 17.75–19) for intermediate and experts (*p* < 0.001, Mann-Whitney U). Interrater reliability was 0.949 (CI 0.983–0.853) for OSATS and 0.945 (CI 0.981–0.842) for Task-Specific Technical Skills. Suggested improvements for the model included the addition of neuro-vascular anatomy and arachnoid mater to simulate bleeding vessels and CSF leak, respectively, as well as improvement in materials to reproduce the consistency closer to that of human tissue and bone.

**Conclusion:**

The TNS Box simulation model has demonstrated face, content, and construct validity as a simulator for the endoscopic endonasal transsphenoidal approach. With the steep learning curve associated with endoscopic approaches, this simulation model has the potential as a valuable training tool in neurosurgery with further improvements including advancing simulation materials, dynamic models (e.g., with blood flow) and synergy with complementary technologies (e.g., artificial intelligence and augmented reality).

## Introduction

Simulation-based training has gained widespread acceptance within neurosurgery over the last decade. This has primarily been driven by restrictions on trainee working hours, increased sub-specialisation and the growing demands to improve patient safety and quality of care (1). These restrictions have meant it has become increasingly challenging to gain wide exposure to neurosurgical procedures, develop surgical skills and achieve the core clinical competencies. These challenges have further been exacerbated by the recent COVID-19 pandemic, which led to a reduction in elective and non-elective cases and subsequently impacted neurosurgical training (2, 3).

Simulation has been proposed as a potential solution to these challenges by allowing for the acquisition and development of technical and non-technical skills in a time-effective manner (4). It provides a safe, realistic, controlled environment where technically demanding tasks can be performed to completion and errors can be made. It permits trainees to enhance their understanding of complex anatomy and reduces the learning curve of surgical procedures (5, 6).

High-fidelity simulation is emerging as a powerful, cost-effective resource within surgical education and has gained growth in sub-speciality areas in neurosurgery, such as aneurysmal surgery (7–9) and neuro-oncology (10). With minimally invasive approaches becoming a standard in the neurosurgical armamentarium, the use of high-fidelity simulation has become an increasingly important method to overcome the steep learning curve associated with these techniques. This is particularly relevant in endoscopic endonasal transsphenoidal surgery due to the complex anatomy, high risk of complications and difficult ergonomics (11, 12). Additionally, there is heterogeneity in the way these procedures are performed which may lead to variation in outcomes (13). As such, dedicated fellowships in endoscopic endonasal surgery are often required to achieve competencies. However, gaining proficiency in endoscopic endonasal techniques requires regular practice and cannot be achieved by observation alone.

There are several simulators available for neuroendoscopic approaches ranging from low fidelity bell pepper trainers to augmented reality (AR)/virtual reality (VR) platforms (14). Many of these have demonstrated validity and utility in improving operative performance (15–18). VR/AR platforms have several advantages within neurosurgery including their low cost, ability to simulate a wide variety of cases and unlimited repetition. However, a drawback of VR/AR simulators is they often lack realistic haptic feedback. More recently, a high-fidelity hybrid (physical and virtual) simulator (UpSurgeOn, TNS Box) has been developed as means to provide a realistic, hands-on experience and improve technical skills in endoscopic endonasal transsphenoidal surgery.

Before implementation as a training tool, however, simulation models require validation to assess their effectiveness. Steps of validation include assessment for realism (face validity), usefulness as a training tool as viewed by experts (content validity) and the ability to differentiate levels of surgical experience (construct validity) (19, 20). At present, there are no validated studies for the UpSurgeOn TNS model. Herein, we aim to determine if the UpSurgeOn TNS Box is a valid education tool for endoscopic endonasal transsphenoidal surgery by assessing its face, content and construct validity.

## Methods

### Model

**Figure 1:**
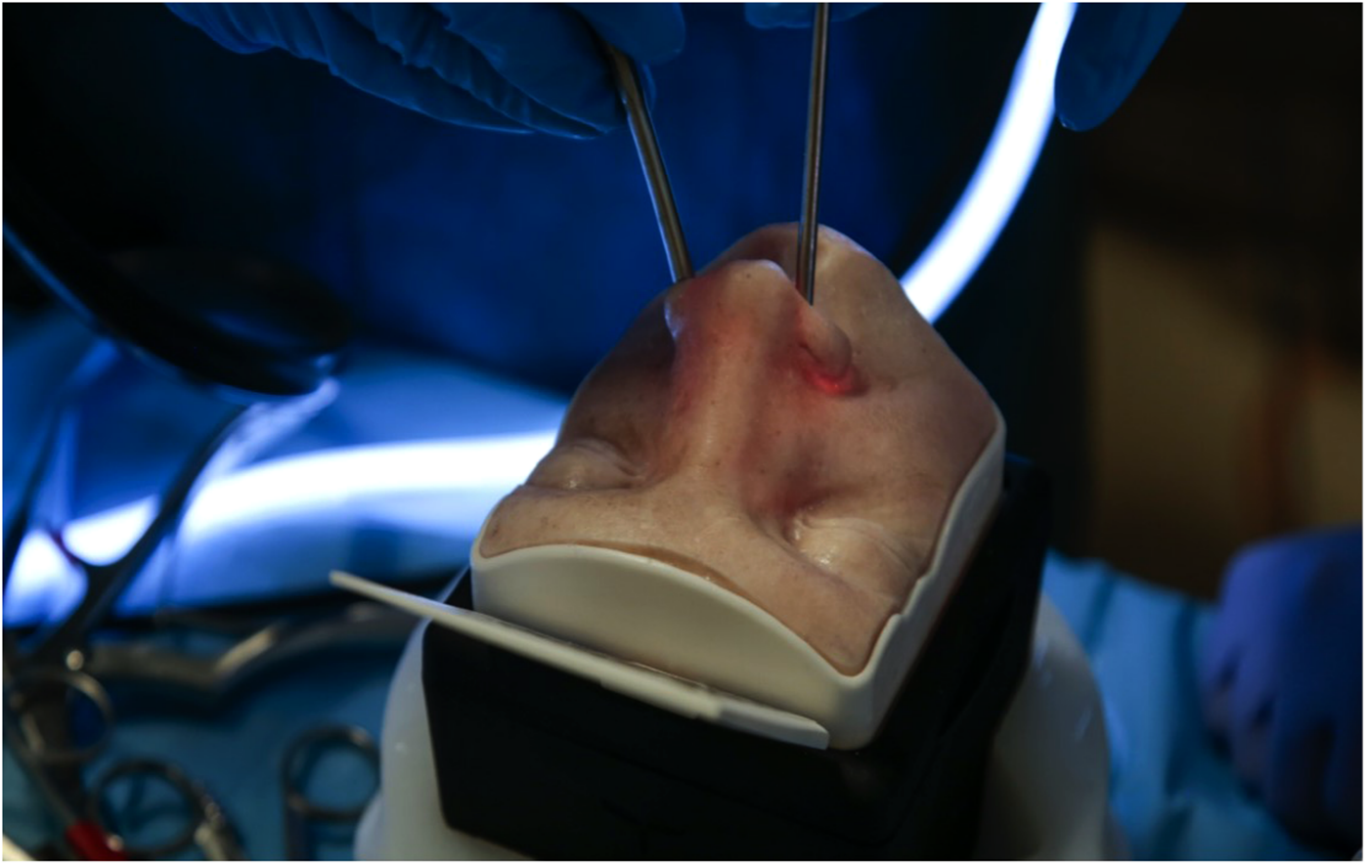
Upsurgeon TNS Box simulator.

**Figure 2:**
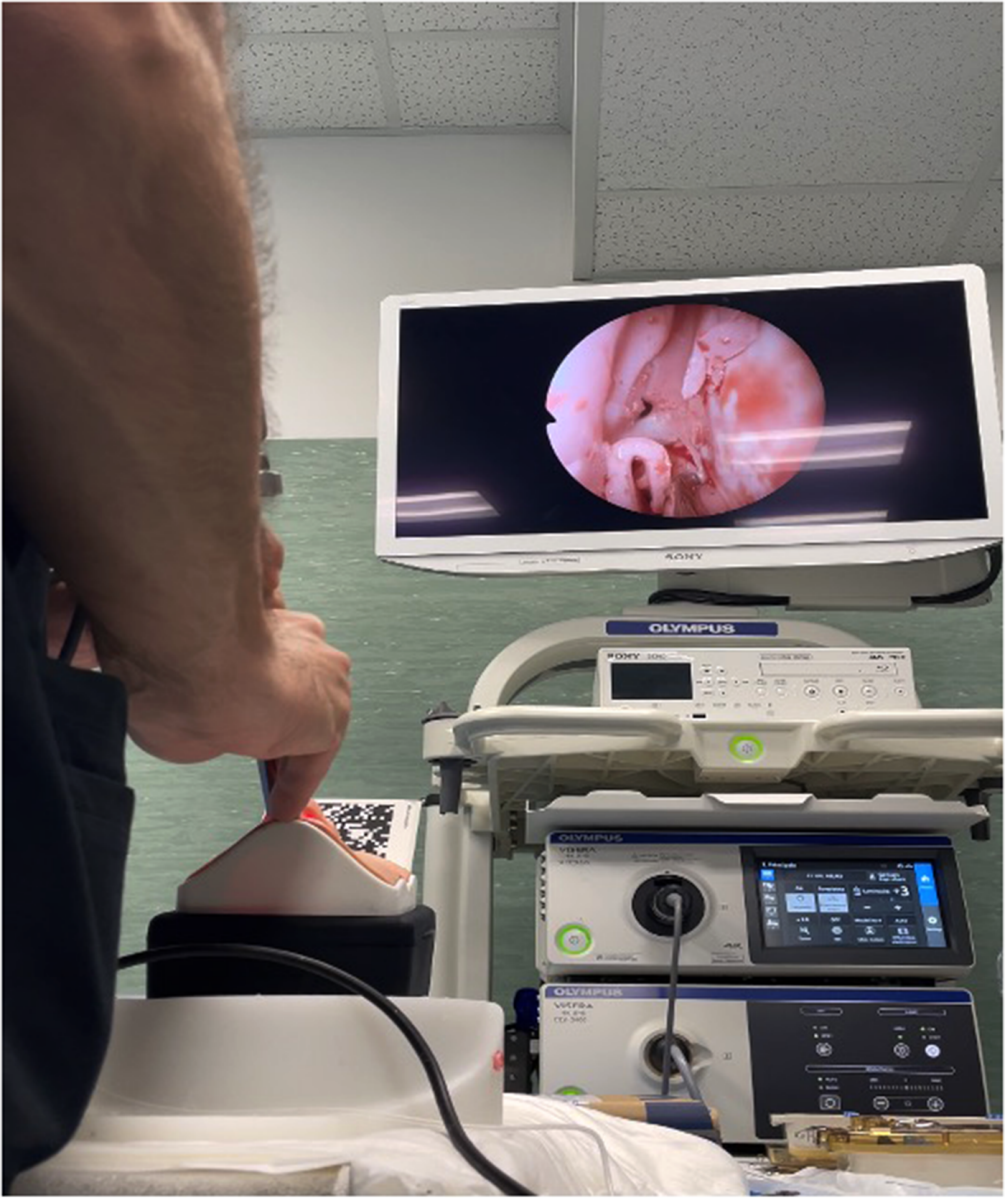
Workstation set up with the UpSurgeOn TNS Box and endoscope.

The Transsphenoidal Box (TNS) is a high-fidelity simulator for the transsphenoidal endonasal approach to the pituitary fossa (Figures 1, 2). The TNS box comprises of a nasal cavity with a 3D face overlay and is manufactured using silicones and resins through 3D printing. The model enables users to explore the nasal cavity, identify anatomical landmarks and resect pituitary tumours endoscopically through careful dissection and drilling techniques.

### Participants

Fifteen participants were enrolled in a prospective, single-centre study. Participants were recruited from multiple neurosurgical centres within the United Kingdom. Subjects were categorised as novice- those in neurosurgical training, or intermediate/experts- senior neurosurgical residents and fellows with prior dedicated pituitary training, and consultants. The novice group had no experience in performing endoscopic transsphenoidal approaches whilst the intermediate and expert participants had varying experience. Participants completed a pre-study questionnaire on demographics and self-report of surgical experience (Appendix 1). Demographic data collected included age, handedness, speciality, stage of training, number of operations observed, assisted and performed, and number of transsphenoidal cases observed, assisted and performed (Tables 1, 2).

**Table 1:** Participant demographics.

	Novice	Intermediate and Expert
Number	10	5
Sex (F : M)	6 : 4	2 : 3
Age median (range)	30 (26–33 years)	39 (33–41 years)
Handedness (R : L)	9 : 1	5 : 0

**Table 2:** Participant experience.

Level	Experience	<5	5–10	11–20	>20
Novice (*n* = 10)	Observed eTSA	8	1	1	0
	Assisted eTSA	9	1	0	0
	Performed eTSA (supervisor scrubbed)	10	0	0	0
	Performed eTSA (supervisor unscrubbed)	10	0	0	0
Intermediate (*n* = 1)	Observed eTSA	1	0	0	0
	Assisted eTSA	0	0	1	0
	Performed eTSA (supervisor scrubbed)	0	0	0	1
	Performed eTSA (supervisor unscrubbed	1	0	0	0
Expert (*n* = 4)	Observed eTSA	0	0	0	4
	Assisted eTSA	0	0	0	4
	Performed eTSA (supervisor scrubbed)	0	0	0	4
	Performed eTSA (supervisor unscrubbed)	0	0	0	4

### Surgical task

Participants were asked to perform a sphenoidotomy using the TNS Box. The task was performed endoscopically using the Olympus S200 visera elite endoscope and participants were supplied with an instrument set. The task focused on the core steps and specific technical skills required during the nasal phase of the eTSA derived from an expert Delphi consensus (13). The four key steps examined included:

(1) identification of the choana, septum and nasal turbinates
(2) lateral displacement of the superior and middle nasal turbinate
(3) ability to identify and assess limits of the sphenoid ostium and
(4) performing an anterior sphenoidotomy appropriately whilst protecting nasoseptal pedicle.

**Figure 3:**
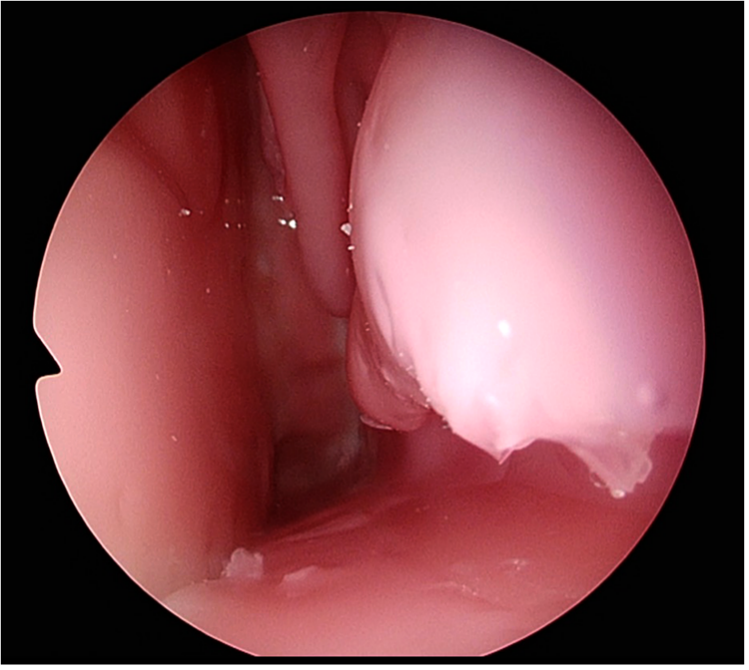
Participant identifying choana and nasal turbinate's on the TNS Box.

**Figure 4:**
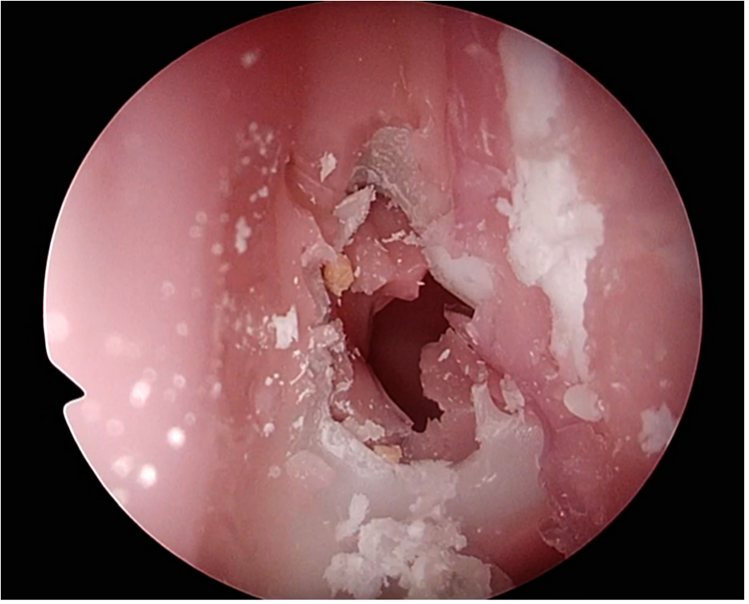
Anterior sphenoidotomy performed on the TNS Box.

Step (1) on the TNS Box is demonstrated in Figure 3 and anterior sphenoidotomy in Figure 4.

### Face and content validity

Intermediate and expert participants completed a post-task questionnaire to assess simulator realism (face validity) and its usefulness as a training tool (content validity) based on a 5-point Likert scale (1 = Strongly Disagree, 2 = Disagree, 3 = Neither Agree nor Disagree, 4 = Agree, 5 = Strongly Agree) (Appendix 2) (28).

### Construct validity

Construct validity was assessed through a post-hoc blinded review of recorded videos by two expert authors. Both expert authors had assisted and performed >150 eTSA procedures and have trainer roles in this sub-speciality. Operative performance was assessed using the modified Objective Structured Assessment of Technical Skills (OSATS) criteria (Appendix 3) (29). Six metrics were assessed, and each was ranked from 1 to 5. A Task-Specific Technical Skill scoring system was generated using published workflow analyses on endoscopic transsphenoidal pituitary adenoma resection (13). Each of the four task steps were ranked from 1 denoting completely inadequate to 5 denoting excellent (Appendix 3).

### Statistical analysis

Median values for OSATS and Task-Specific Technical skills were analysed for statistical differences between groups using Mann-Whitney U test. Analysis was performed using GraphPad (GraphPad Software Inc, California, USA) and SPSS Version 28.0 (IBM, UK). Results with a *p*-value of <0.05 were considered a statistically significant difference.

## Results

### Participants

Fifteen participants (10 novices, 1 intermediate and 4 experts) completed the surgical task. The novice group consisted of 10 neurosurgical trainees ranging from year one of training to year six and had minimal endoscopic pituitary experience. The intermediate and expert group were combined and consisted of one consultant neurosurgeon, three senior clinical pituitary fellows and one senior resident all of whom had dedicated sub-speciality experience. The median age of the novice group was 30 years (range: 26–33 years), whereas the median age of the intermediate and expert group was 39 years (range: 33–41 years). The group consisted of 8 females (53.3%) and 7 (46.7%) males. Fourteen participants (93.3%) were right-handed and one participant (6.7%) was left-handed (Table 1).

### Face and content validity

All intermediate and expert participants (*n* = 5, 100%) completed the post-task questionnaire. Sixty percent (*n* = 3) of intermediate and expert participants agreed that the TNS Box was realistic, whilst forty percent (*n* = 2) were neutral. Intermediate and expert participants strongly agreed (*n* = 3, 60%) or agreed (*n* = 2, 40%) that the TNS Box was useful as a training tool.

### Construct validity

**Figure 5:**
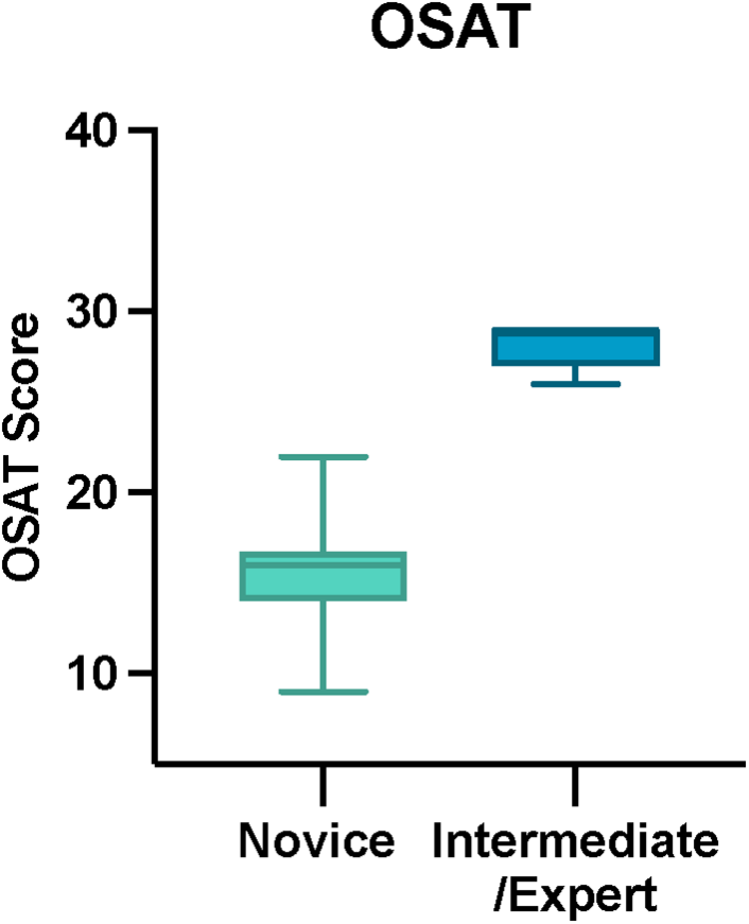
Total OSAT score for novice and intermediate and expert participants. The horizontal solid line in the middle of the box is the median value of the scores and the lower and upper boundaries indicate the 25th and 75th percentiles, respectively. The largest and smallest observed values are shown; lines (whiskers) are drawn from the ends of the box to those values.

**Figure 6:**
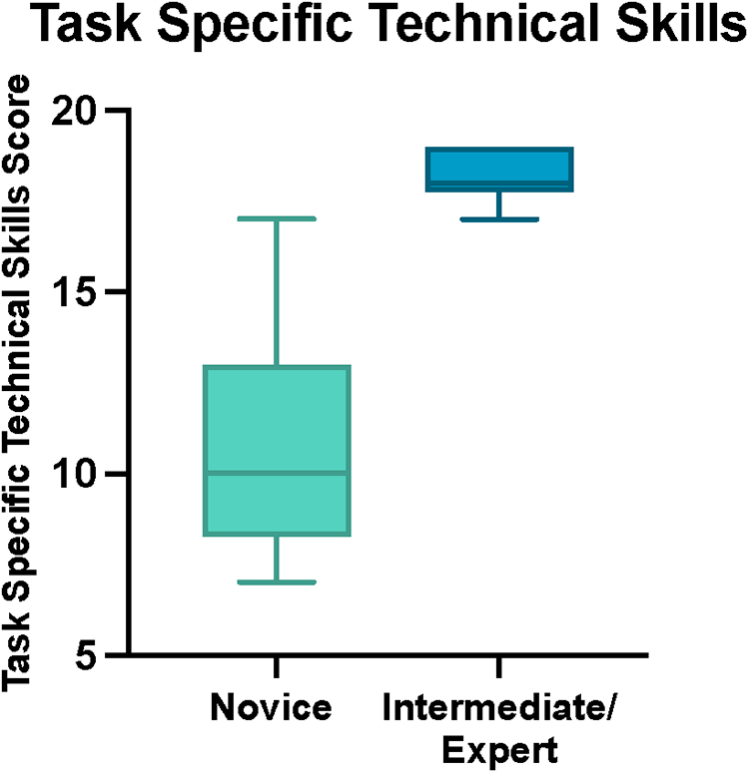
Total task specific technical skills score for novice and intermediate and expert participants. The horizontal solid line in the middle of the box is the median value of the scores and the lower and upper boundaries indicate the 25th and 75th percentiles, respectively. The largest and smallest observed values are shown; lines (whiskers) are drawn from the ends of the box to those values.

The total OSATS and Task-Specific Technical Skills scores were calculated for each participant. Median total OSATS score was 16/30 (IQR 14–16.75) for novices and 29/30 (IQR 27–29) for intermediate and experts (*p* < 0.001, Mann-Whitney U) (Figure 5). Median Task-Specific Technical Skills score was 10/20 (IQR 8.25–13) for novices and 18/20 (IQR 17.75–19) for intermediate and experts (*p* < 0.001, Mann- Whitney U) (Figure 6). Six of ten novices did not complete sphenoidotomy whilst all intermediate and experts completed the task.

### Interrater reliability

Interrater reliability was used to assess the extent to which the two raters agreed. Interrater reliability was 0.949 (CI 0.983–0.853) for OSATS and 0.945 (CI 0.981–0.842) for Task-Specific Technical Skills.

### Qualitative feedback

Intermediate and experts were asked to provide feedback on the TNS model. Responses focused on suggested improvements including making the models dynamic by introducing CSF and blood as well as pulsatile vessels. Other responses focused on the materials used suggesting changes to bone to make it less thick as well as changes to soft tissue including the mucosa and tumour. The mucosa was described as too elastic whilst the tumour was too firm and not in keeping with that of human tissue.

## Discussion

### Principal findings

In this study, we sought validation of the TNS Box for the endoscopic endonasal transsphenoidal approach. The TNS Box was validated according to 3 components: face, content and construct validity. Face validity is a subjective measurement used to determine if the model provides a realistic demonstration of the task. In the context of surgical simulation validation, expert opinions are sought to provide a reliable measure of face validity. This is, in part, due to the increased level of familiarity with the operative condition. Therefore, face validity of the TNS Box in our study was determined by assessing the responses of the intermediate and expert neurosurgeons. Responses from intermediate and experts were variable, with three agreeing the model was realistic and two remaining neutral. On this basis, the TNS Box showed moderate face validity.

A goal of simulation is to closely reproduce the advantages offered by cadaveric training. However, formulating materials to mimic real tissue and reproducing the haptics of the procedure can often be challenging. Although realism of the simulator may be useful for simulation-based training in surgery, several studies have shown the degree of fidelity appears to be independent of educational effectiveness, providing the model meets the functional requirements of the clinical task (6, 30, 31). Other studies have also shown the effect of simulator fidelity depends upon the experience of the user and goals of the learner (32), where low-fidelity simulation is more suitable for initial learning and performance improvement, and high-fidelity simulation more suitable for the transfer of skills and assessment (33). In our study, the TNS Box showed high content validity with all intermediate and experts confirming its utility as a training tool for the eTSA approach. Furthermore, nine of ten novices ranked the TNS Box as a useful learning tool.

Finally, an important consideration when validating a simulation model is its ability to discriminate between different levels of surgical proficiency. For a simulator to have construct validity, experts must outperform novices during standardised simulated tasks. In our study, this was based on performance scores which considered several parameters including technical skills and ability to perform sphenoidotomy. Six of ten novices were unable to perform opening of the sphenoid ostium, whilst all intermediate and experts performed this task. The TNS model showed a high construct validity with statistical differences in both the OSATS and Task-Specific Technical Skills score between the novice and the intermediate and expert groups. The construct validity findings were further supported by high interrater reliability.

Whilst the TNS Box has demonstrated face, content and construct validity, it does however have several limitations. At present, it lacks important neurovascular anatomy including the carotid arteries and sphenopalatine arteries. These are important structures to be aware of whilst undertaking this approach. Another limitation is the type of material used. Compared to human tissue, the mucosa is more elastic and the sella is thicker requiring a drill to access the tumour. As well as that, the tumour is too firm. Due to these limitations, phases beyond the nasal phase of the procedure were not evaluated. These aforementioned features could be addressed in order to reproduce the consistency close to that of human soft tissue and bone and thus enabling evaluation of the sphenoid, sellar and closure phases. Additional features to make the simulator more sophisticated could be the addition of arachnoid mater in order to simulate CSF leak.

### Findings in context of existing literature

Endoscopic endonasal transsphenoidal surgery is a technically demanding operation with a steep learning curve. The procedures tend to be low volume but require a high number to achieve surgical proficiency. As the use of this approach to treat sellar and suprasellar lesions continues to expand, there becomes a greater need for the use of training tools to enhance the understanding of anterior skull base anatomy and advance neuroendoscopy skills.

There are several challenges inherent to the endoscopic endonasal transsphenoidal approach which make the use of simulation models an ideal platform to develop surgical techniques. Firstly, the endoscope can be technically challenging to use due to its limited angulation and manoeuvrability. Secondly, endoscopic transsphenoidal approach (eTSA) to the anterior skull base often requires extensive bone work adjacent to critical neurovascular structures. Thirdly, access to the area is through a narrow surgical corridor. Limited space, due to the presence of the endoscope and surgical instruments in the field, creates ergonomic challenges. Simulation models enable the trainee to develop these technical skills and gain familiarity with the instruments used. They enable performance feedback in a safe environment and have the potential to create and develop skills transferrable to the operating theatre.

A variety of simulators for training in endoscopic skull base surgery have been produced (Table 3). Cadaveric models have been used to simulate injury to the internal carotid artery (ICA), a rare but life-threatening complication of the eTSA. Donoho et al. perfused cadavers with artificial blood and demonstrated face and construct validity, as well as utility as an educational tool (23, 34). Other cadaveric studies have looked at simulating CSF leak repair and demonstrated face, content and construct validity (24).

**Table 3:** Examples of current simulation models for the endoscopic endonasal approach.

Simulation model	Procedure	Study
Task trainers/3D models	Endoscopic endonasal drilling techniques	Tai et al., 2016 (18)
	Pre-operative planning for pituitary adenoma resection	Huang et al., 2019 (21)
	Endoscopic transsphenoidal pituitary adenoma resection	Okuda et al., 2011 (22)
Cadaveric trainers	ICA injury	Donoho et al., 2019 (23)
	CSF leak repair	AlQahtani et al., 2020 (24)
	Tumour resection	Gagliardi et al., 2018 (25)
VR/AR models	NeuroTouch: endoscopic endonasal transsphenoidal approach	Rosseau et al., 2013 (17)
	Endoscopic endonasal transsphenoidal approach	Kim et al., 2020 (26)
	Pre-operative planning	De Notaris et al., 2014 (27)

Advances in 3D printing have allowed for simulation models to be created for complex surgical pathology with adequate tactile feedback. Studies have demonstrated their use in anatomic education (35) and pre-operative planning of endoscopic endonasal transsphenoidal surgery. Huang et al. showed the potential of 3D printing to demonstrate adjacent anatomical relationships and aid planning techniques for tumour resection (21). Other studies have used 3D models to improve eTSA technical skills including drilling techniques, curetting, aspiration and navigation (36, 37). Although these models have shown potential application, they have not undergone validation. Other efforts at the simulation of endoscopic endonasal skull base surgery have included VR and AR simulation models (17, 26). Although VR/AR has the potential as an effective educational tool, limitations in haptic feedback remain a major concern.

In the future, surgical simulations will become more sophisticated through the integration of high-fidelity haptic feedback. This will offer several advantages including improved tissue manipulation, better spatial awareness and a reduction in surgical complications (38). The integration of AR into high-fidelity simulators has been a promising new development. AR overlays have the potential to simulate variations in surgical anatomy, illustrate various surgical approaches and develop complex surgical skills (39). Furthermore, this provides a good solution for high-fidelity neurosurgical training in low-middle income countries (40). The application of AI to surgical simulation may prove to be another useful addition by providing objective feedback on performance and differentiating between skill levels (41). Studies have shown its potential for improving operative efficiency and patient outcomes through its ability to recognise stages of the procedure, provide guidance on instruments required and identify important anatomic relations (42).

### Strengths and limitations

There are several strengths to our study. Construct validity was assessed using the OSATS score which is a well-established tool used to assess technical skills. This allowed for a valid and reliable assessment of the participant's surgical skills. The task assessed was derived from an expert Delphi consensus on the phases and core steps for endoscopic transsphenoidal pituitary adenoma resection (13). This provided a framework to assess knowledge of the approach and surgical ability. Operative videos were assessed blindly by two expert neurosurgeons. The inter-rater reliability was high suggesting a high degree of agreement between the two examiners.

This study however has several limitations. Firstly, we did not assess predictive validity. This would be a useful measure to determine improvement in skills and performance in the operative theatre. However, this concept is difficult to evaluate due to patient safety and ethical concerns. Secondly, the AR overlay function for the TNS Box was not used in our study in order to simplify the task. However, this is a useful addition to the model which can be used to simulate variations in anatomy and demonstrate various surgical approaches. Thirdly, the number of participants involved in the study was rather low, however this is in keeping with existing validation studies (43). In addition, we combined intermediate and expert surgeons within our study. This meant there was some variation in surgical procedures observed, assisted and performed. However, several studies within the literature support this method for validation studies (43). Lastly, the model had a few limitations including the types of material used making it largely inconsistent with that of human tissue, the lack of bleeding vessels and important neurovascular structures, and the lack of arachnoid mater to simulate CSF leak. As a result of the unrealistic consistency of the tumour and the sphenoid sinus anatomy, only the nasal phase of the procedure was examined. With the iterative improvement of the TNS Box, the sphenoid, sellar and closure phases can be evaluated.

## Conclusions

This present study demonstrates face, content and construct validity for the TNS Box and highlights the model as a potentially useful surgical skills training tool for the eTSA approach. Further improvements include advancing simulation materials, dynamic models (e.g., with blood flow), synergy with complimentary technologies (e.g., AI and AR), and integration as a supplement to modern surgical curricula.

## Data Availability

The raw data supporting the conclusions of this article will be made available by the authors, without undue reservation.

## Appendix 1 Pre-study questionnaire on the demographics of each participant

Name:

Age:

Handedness:

Speciality:

Stage of training:

Number of neurosurgical cases (of any sort)

(a) Observed
(b) Assisted
(c) Performed

Number of transsphenoidal cases (of any sort)

(a) Observed
(b) Assisted
(c) Performed

## Appendix 2 Post-study questionnaire examining the face and content validity of the TNS Box

Face Validity

The model was realistic?

□ Strongly Agree
□ Agree
□ Neutral
□ Disagree
□ Strong Disagree

Content Validity

The model was useful?

□ Strongly Agree
□ Agree
□ Neutral
□ Disagree
□ Strongly Disagree

## Appendix 3 Score sheet used to assess construct validity of the TNS Box

**Table:**

Task: Perform nasal approach & access the sphenoid sinus
Objective Structured Assessment of Technical Skills
Respect for tissue
1. Frequently used unnecessary force on tissue or caused damage by inappropriate use of instruments
5 Consistently handled tissue appropriately with minimal damage
Time and motion
1. Many unnecessary moves
5. Clear economy of movement and maximum efficiency
Instrument handling
1. Repeatedly makes tentative or awkward moves with instruments
5. Fluid moves with instruments and no awkwardness
Flow of operation
1. Frequently stopped operating or needed to discuss next move
5. Obviously planned course of operation with efficiency from one move to another
Knowledge of instruments
1. Frequently asked for wrong instrument or used inappropriate instrument
5. Obviously familiar with the instruments and their names
Knowledge of procedure
1. Insufficient knowledge. Looked unsure and hesitant
5. Demonstrated familiarity with all steps of the operation
Task Specific Technical Skills
Able to identify choana, septum and turbinates
1: Completely inadequate
5: Excellent
Effective lateral displacement of middle turbinate and superior turbinate
1: Completely inadequate
5: Excellent
Able to identify and assess the limits of the sphenoid ostium
1: Completely inadequate
5: Excellent
Performs anterior sphenoidotomy appropriately whilst protecting nasoseptal pedicle
1: Completely inadequate
5: Excellent
